# Detection of *Fusarium* Species in Clinical Specimens by Probe-Based Real-Time PCR

**DOI:** 10.3390/jof5040105

**Published:** 2019-11-12

**Authors:** Jan Springer, Grit Walther, Volker Rickerts, Axel Hamprecht, Birgit Willinger, Daniel Teschner, Hermann Einsele, Oliver Kurzai, Juergen Loeffler

**Affiliations:** 1 Department for Internal Medicine II, University Hospital of Wuerzburg, 97080 Wuerzburg, Germany; einsele_h@ukw.de (H.E.); loeffler_j@ukw.de (J.L.); 2 National Reference Center for Invasive Fungal Infections (NRZMyk), Leibniz Institute for Natural Product Research and Infection Biology, Hans Knöll Institute, 07745 Jena, Germany; Grit.Walther@hki-jena.de (G.W.); or okurzai@hygiene.uni-wuerzburg.de (O.K.); 3 Robert Koch Institute Berlin, 13353 Berlin, Germany; RickertsV@rki.de; 4 Institute for Medical Microbiology, Immunology and Hygiene, University Hospital of Cologne, University of Cologne, 50935 Cologne, Germany; axel.hamprecht@uk-koeln.de; 5 Institute for Medical Microbiology and Virology, Carl von Ossietzky University of Oldenburg, 26129 Oldenburg, Germany; 6 Division of Clinical Microbiology, Institute of Hygiene and Medical Microbiology of the University, Vienna University Hospital, 1090 Vienna, Austria; birgit.willinger@meduniwien.ac.at; 7 Department of Hematology, Medical Oncology, and Pneumology, University Medical Center of the Johannes Gutenberg University, 55122 Mainz, Germany; Daniel.Teschner@unimedizin-mainz.de; 8 Institute for Hygiene and Microbiology, University Wuerzburg, 97080 Wuerzburg, Germany

**Keywords:** probe-based real-time PCR, Fusarium, bronchoalveolar lavage fluid, fungal molecular diagnostics

## Abstract

The mold *Fusarium* is a ubiquitous fungus causing plant, animal and human infections. In humans, *Fusarium* spp. are the major cause of eye infections in patients wearing contact lenses or after local trauma. Systemic infections by *Fusarium* spp. mainly occur in immunosuppressed patients and can disseminate throughout the human body. Due to high levels of resistance to antifungals a fast identification of the causative agent is an urgent need. By using a probe-based real-time PCR assay specific for the genus *Fusarium* we analysed several different clinical specimens detecting *Fusarium* spp. commonly found in clinical samples in Germany. Also, a large collection of lung fluid samples of haematological patients was analysed (*n* = 243). In these, two samples (0.8%) were reproducibly positive, but only one could be confirmed by sequencing. For this case of probable invasive fungal disease (IFD) culture was positive for *Fusarium* species. Here we describe a rapid, probe-based real-time PCR assay to specifically detect DNA from a broad range of *Fusarium* species and its application to clinically relevant specimens.

## 1. Introduction

The mold *Fusarium* is a ubiquitous fungus well known as a plant pathogen and mycotoxin producer [1]. There are more than 300 phylogenetically distinct species known [2]. At least 70 are involved in invasive fusariosis. Within these, the *Fusarium solani* species complex (FSSC) is responsible for the most fusariosis cases [3,4], followed by other complexes causing human infections (*Fusarium oxysporum* species complex (FOSC), *Fusarium dimerum* species complex (FDSC), *Fusarium fujikuroi* species complex (FFSC; also known as *Gibberella fujikuroi* species complex GFSC), and *Fusarium incarnatum–Fusarium equiseti* species complex (FIESC)) [3]. Invasive fusariosis is a life-threatening invasive fungal disease (IFD) with poor outcome that mostly occurs in immunocompromised patients [5] with mainly haematological malignancies [6]. Improved fungal diagnostics and treatment has led to a decrease in the number of infections caused by *Candida* and *Aspergillus,* but also to a rise of so-called rare fungal pathogens, including members of the genus *Fusarium* [7,8,9,10]. As *Fusarium* may display high levels of resistance to most classes of antifungals [11], a fast identification of the causative agent is mandatory for effective treatment leading to a good patients’ outcome. The drug recommended for first-line treatment is voriconazole [12].

To improve *Fusarium* identification, DNA detection via polymerase chain reaction (PCR) is a rapid and sensitive tool. There are different methods to detect *Fusarium* by PCR including conventional and real-time (hybridization or hydrolysis probe based) PCR [6,13,14,15], which are limited by their high turnaround time, detection of some selected species, or uncommon real-time PCR formats. Here we describe a rapid, probe-based real-time PCR assay to specifically detect DNA from a broad range of *Fusarium* species and its application to clinically relevant specimens.

## 2. Materials and Methods

### 2.1. DNA from Clinical Specimens Positive for Fusarium spp. and Isolates

From 1998 to 2017, clinical specimens were sent to German laboratories to detect fungi. Fungal DNA extracts of clinical samples (*n* = 10) or clinical isolates (*n* = 12) containing DNA of *Fusarium* species were kindly provided (National Reference Center for Invasive Fungal Infections, Jena, Robert Koch Institute, Berlin, and University Hospital of Cologne, Institute for Medical Microbiology, Immunology and Hygiene) reflecting the most common causative fungi isolated from disseminated and local infections [16]. Samples comprised several different *Fusarium* spp. (*F. dimerum complex*, *n* = 2; *F. keratoplasticum*, *n* = 2; *F. musae*, *n* = 2; *F. oxysporum* complex, *n* = 5; *F. petroliphilum*, *n* = 6; *F. proliferatum*, *n* = 2; *F. sacchari*, *n* = 1; *F. verticilloides*, *n* = 1). Twelve were related to the eye (contact lens fluid, cornea tissue, eye fluid), 5 were derived from blood cultures, 2 from bronchoalveolar lavage fluid (BALF), and 3 from skin or mucosal tissue (sinus).

### 2.2. Bronchoalveolar Lavage Fluid (BALF)

From July 2016 to December 2018, BALF samples taken for routine clinical investigation of chest infection (new or progressive lung infiltrates) in adult patients (≥ 18 years old) with haematological malignancies (HM) across ten hospitals, 9 centers from Germany and one from Austria, were retained. DNA eluates of BALF samples were stored at −20 °C for retrospective PCR testing. In addition to standard of care procedures, each sample was tested retrospectively for the presence of fungal DNA by a newly developed *Fusarium*–specific real-time PCR. Patients were categorized according to revised European Organization for the Research and Treatment of Cancer (EORTC)/Mycoses Study Group (MSG) criteria [17] resulting in 7 proven IFD (3.2%; *Aspergillus*, *n* = 3; *Rhizopus*, *n* = 1; not defined, *n* = 3), 72 probable IFD (32.6%), 108 possible IFD (48.9%), 29 undetermined IFD (13.1%), and 5 unknown (2.3%). Bronchoscopy was performed in accordance with institutional protocols. The study was approved by the local ethics committees of the UKW (Ethikommission des Universitätsklinikums Würzburg, 270/15), and elsewhere as appropriate.

### 2.3. DNA Extraction from BALF

All steps for extracting DNA from BALF were performed in a class II laminar-flow cabinet. Briefly, samples were vortexed, and 0.5 ml of BAL fluid was centrifuged at 5000× *g* for 7 min. The supernatant was transferred to a new tube. The remaining pellet was bead-beaten after adding 50 µL of BALF supernatant for 90 secs using 1.4-mm (diameter) ceramic beads. Using a commercial extraction kit (High Pure template preparation kit, Roche) both fractions were combined and extracted as described before [18]. Elution volume was adjusted to 70 µL.

### 2.4. 28S-Based Fusarium-Specific Real-Time PCR

The 28S-based assay described by Muraosa et al. [13] was adapted by designing a specific hydrolysis probe including the originally used nucleotides. The original reverse primer was modified in one nucleotide (5-GCTATCGGTCTCTGGCC**R**G-3; R for G or A) and a novel locked nucleic acid (LNA nucleotides in bold and brackets) probe (Sigma Life Science; qFUS-LNA76 5- FAM-CC[G][T]CT[G][G]T[T][G]GA-BHQ-3) was designed. Where possible, DNA eluates were analysed in duplicate (226 BALF, 16 others). Amplifications were performed in a 20 µL mixture using a StepOnePlus thermocycler (Applied Biosystems). Each reaction mixture contained 10 µL 2× Takyon Rox Probe MasterMix UNG (Eurogentec), 450 nM forward and reverse primer, 150 nM probe, and 5 µL of extracted DNA. The assay, described by Muraosa et al., was specific for *Fusarium* confirmed by testing other fungi including *Aspergillus* and *Candida* [13]. Amplification was carried out applying a standard thermocycling protocol: Uracil-DNA glycosylase (UNG) activation was at 50 °C for 2 min, and initial *Taq* polymerase activation was at 95 °C for 3 min, followed by 50 cycles of 95 °C for 10 s, 55 °C for 30 s, and 72 °C for 20 s (standard ramp speed and standard cycling conditions). In each run, negative (molecular grade water) and positive controls (DNA of *Fusarium* sp.) were included. Amplicons of clinical samples were purified, sequenced (the classical dye chain-termination sequencing method (“Sanger sequencing”) by a commercial company (LGC, Berlin)), and analyzed by using BLAST analysis (National Center of Biotechnology Information, Washington DC, www.ncbi.nlm.nih.gov/BLAST) as described before [19].

To determine the limit of detection (LoD) of qPCR assay, DNA was measured using a NanoDrop (ND-1000, Peqlab) and serially diluted. Exemplarily, the LoD using DNA of *F. solanii* was determined to be 15 fg, fitting perfectly to the originally described LoD (one copy of standard DNA which corresponds to approximately 30 fg) [13]. PCR efficiency was 91.1% using 5 ten-fold dilutions in triplicates ranging from 1.2 to 12,000 copies.

## 3. Results

### 3.1. Clinical Specimens Positive for Fusarium spp.

In total, 22 DNA eluates from clinical isolates (*n* = 12) and clinical samples (*n* = 10) were tested for the presence of fungal DNA. In 20 eluates comprising a variety of *Fusarium* spp., DNA of the genus *Fusarium* could be detected (*F. dimerum* complex, *F. keratoplasticum*, *F. musae*, *F. oxysporum* complex, *F. petroliphilum*, *F. proliferatum*, *F. sacchari*, *F. verticilloides*). All *Fusarium* spp. commonly found in clinical samples in Germany [16] could be detected by using this real-time PCR assay. Two of 10 clinical samples were not detected. For one negatively tested sample, the eluate volume was limited and no further testing was possible, neither for a duplicate using the *Fusarium*-specific PCR assay nor for testing of PCR inhibition. The second negative sample was reproducibly negative and showed no PCR inhibition. Interestingly, both originally contained *F. oxysporum* complex as determined by not-yet-published PCR assays (personal communication, G. Walther), which can be generally detected by our PCR assay as shown by testing other specimens (*n* = 3). Unsuitable storage or transport conditions of tested DNA samples cannot be excluded as a reason for PCR negativity.

### 3.2. Bronchoalveolar Lavage Fluid (BALF)

In total, 243 BALF samples from 221 patients were collected and retrospectively analyzed for the presence of fungal DNA. Two samples (0.8%) showed positive PCR results for *Fusarium* DNA, one possible (Cq mean 33.8) and one probable IFD case (Cq mean 24.2). Sequencing revealed the genus *Fusarium* for the probable IFD case, but no homology to fungi was found for the possible case using forward or reverse primer for sequencing. Interestingly, the probable case was also positive for GM testing and *Aspergillus*-specific PCR, suggesting a fungal coinfection. This patient was a 53-year-old man suffering from non-Hodgkin lymphoma (diffuse large B-cell). Because of a relapse, he was treated with irutinib (very good partial remission). Two months later, he was admitted for stem cell transplantation (SCT). Three weeks after transplantation, pulmonary aspergillosis was diagnosed because of positive imaging and GM detection (serum). However, *Aspergillus* sp. was never detected in any of the multiple cultures (blood and BALF), but *Fusarium* species was cultured from BALF. The patient was treated successfully with voriconazole, and GM levels decreased under the cut-off (0.5) within two weeks of treatment. A possible double infection of *Aspergillus* and *Fusarium* could have been treated successfully by administering voriconazole, as both pathogens are often susceptible to this antifungal drug.

The possible IFD case was a 66-year-old male patient with mantle cell lymphoma scheduled for a high-dose (HD) chemotherapy with autologous SCT (aSCT). Initially, he presented with a good performance status with no significant clinical findings. After HD chemotherapy with rituximab, bis-*chloroethyl*-nitrosourea (BCNU), etoposide, cytarabine, and melphalan, the patient received aSCT and developed neutropenia. On day 5 after aSCT, fever occurred and empirical antibiotic treatment with piperacillin/tazobactam was initiated. However, the patient still remained febrile and consequently a pulmonary CT scan of the lung was performed showing signs of atypical pneumonia triggering antifungal treatment with voriconazole. A bronchoscopy was performed and BALF was found to be positive for Herpes simplex virus, *Escherichia coli*, and *Pseudomonas aeruginosa*. After adapting the antibiotic treatment to imipenem according to the microbiological susceptibility testing, the patient recovered and became afebrile within two days. A fungal infection seems therefore unlikely, but we cannot exclude it.

## 4. Discussion

PCR is an established, very sensitive molecular tool, which has shown its potential to detect fast and specific pathogens in many fields, leading to earlier diagnosis and improved patient outcome. Here we describe the application of a *Fusarium*-specific probe-based real-time PCR assay to clinical specimens. This assay was able to detect all *Fusarium* species commonly found in clinical samples derived from Germany [16].

In our high-risk cohort for IFD (HM patients with lung infiltrates), we found two BALF samples (0.8%) containing DNA of *Fusarium* spp. One probable IFD case also showed signs for invasive aspergillosis (positive GM assay in serum). The second case (possible IFD) could not be confirmed on sequence level. Similar frequencies of *Fusarium* in BALF were recently described by two studies, ranging from 0.6 to 2% [20,21]. Because of this low frequency, clinical diagnostic studies regarding pulmonary fusariosis are difficult to perform, as huge patient numbers are necessary. Both studies used a broad-range PCR to detect fungal DNA, meaning that *Fusarium* is only one detectable fungus beside many others. One could speculate that specific PCR assays are more sensitive than broad-range ones [22], but also the type of investigated patient cohort and the region play a role. This concerns the fungal distribution on genus level, but also on the species level. FSSC is described to be responsible for the most cases of invasive fusariosis in Japan (73%; [4]) and the United States (60%, [23]), but not in Turkey with 42% [24], where FFSC is the most reported *Fusarium* complex (52%). Similar results are reported for the epidemiology of *Aspergillus terreus*, as this fungus is usually accusing a small proportion of invasive aspergillosis while in specific regions the detection rate is much higher [25].

As sequencing of the possible IFD patient could not confirm the presence of *Fusarium* DNA and also the clinical course was not typical for an IFD, the major involvement of a fungal pathogen is unlikely in this patient. However, in the probable IFD patient different fungal biomarkers were found (culture, GM, PCR), increasing the probability of IFD. As even biomarkers of two different fungal species were detected which can be easily performed by specific molecular tools, e.g. real-time PCR assays, a co-infection of *Aspergillus* and *Fusarium* in this patient is likely. In consequence, the number of co-infections may be underestimated in general, as other studies reported up to 33% of fungal co-infections, which is especially described for *Aspergillus* and Mucorales [22,26,27,28]. GM testing is described to detect *Aspergillus*, but also to cross-react with other fungi including *Penicillium*, *Geotrichum*, yeasts, and *Fusarium* [29]. Therefore, a positive GM test could indicate the presence of *Aspergillus* but also of *Fusarium*.

Using the described qPCR assay enables fast and specific identification of *Fusarium*, but only on genus level; for some authors, identification at species level is necessary [30], whereas others stated that in clinical practice, species identification is not necessary to find suitable treatment options [3]. A positive test result could be used to trigger or re-evaluate an antifungal treatment regimen because all members of the order *Fusarium* show intrinsic resistances. Therefore, remaining treatment options are limited and should be applied as early as possible. In contrast to our assay, previously described assays to detect *Fusarium* DNA have some disadvantages. Conventional PCR assays are highly prone to contamination, are time-consuming, and cannot quantify fungal load, whereas real-time assays are performed in a closed system, minimizing contamination risk (no necessity to handle amplicons to perform a second, nested PCR assay or to visualize PCR results by gel electrophoresis). Furthermore, quantification is possible and monitoring of *Fusarium* DNA, e.g., in serum samples, could guide treatment effectively and improve patients’ outcome.

As fusariosis is a rare disease and is caused by many different species, large future studies have to evaluate this issue by screening many clinical samples with a highly sensitive method. Pretest probability can be increased by analyzing high risk patient cohorts or specimens directly taken from the focus of the disease.

## 5. Conclusions

Here we describe a rapid, probe-based real-time PCR assay to specifically detect DNA from a broad range of *Fusarium* species and a first pilot application to clinical samples. Validation of positive PCR assays should be confirmed by sequencing. The occurrence of fungal co-infections might be underestimated. This assay is most suitable to confirm the diagnosis of fusariosis.
